# Influence of dietary fiber intake and soluble to insoluble fiber ratio on reproductive performance of sows during late gestation under hot climatic conditions

**DOI:** 10.1038/s41598-022-23811-8

**Published:** 2022-11-17

**Authors:** Joseph Moturi, Abdolreza Hosseindoust, Habeeb Tajudeen, Jun Young Mun, Sang Hun Ha, Jin Soo Kim

**Affiliations:** Department of Animal Industry Convergence, Kangwon National University, Chuncheon, 24341 Republic of Korea

**Keywords:** Biochemistry, Biological techniques, Cell biology, Immunology, Physiology

## Abstract

This study evaluated dietary fiber (DF) level and the ratio of soluble fiber (SF): insoluble fiber (ISF) impact on sows’ reproductive performance under heat stress. Forty sows at day 90 of gestation were assigned to four treatments. HH diet had, 20% DF, 1:4, SF:ISF ratio; HL, 20% DF, 1:6, SF:ISF ratio; LH, 14% DF, 1:4, SF:ISF, LL, 14% DF, 1:6, SF:ISF. Results showed that; lactation back-fat loss was lower (P < 0.05) in HH . Feed intake was higher (P < 0.05) in HH and HL. Farrowing duration shorter (P < 0.05) in HH. Constipation index was higher (P < 0.05) in HH and LH. Weaning piglets’ body weight was greater (P < 0.05) in HH than LH and LL. Hair cortisol was lower (P < 0.05) in HH than HL, and LL. Acetate, propionate, isovalerate, and butyrate was higher (P < 0.05) in HH and LH. Plasma zonulin, fecal lipocalin-2 were lower (P < 0.05) in HH, and HL. Superoxide dismutase tended to be higher (P = 0.056) and malondialdehyde tended to be lower (P = 0.069) in HH and HL. We opined that higher levels of dietary fiber and soluble fiber could ameliorate heat stress in gestating sows.

## Introduction

The elevated ambient temperatures during summer negatively impacts on average daily feed intake (ADFI), which in turn adversely affects physiological processes and reproductive performance of sows^1,2^. This occurs when blood flow is diverted from visceral organs to the skin in order to dissipate heat. Limited blood flow to the uterus and the ovary may lead to utero hypoxia, and insufficient ovarian function. Sows are particularly susceptible to extreme heat during their gestation and lactation periods. When exposed to temperatures over their thermal neutral zone (25–35 °C), sows exhibit unfavorable reproductive consequences, including anestrus, longer inter-estrus intervals prolonged weaning to estrus intervals, low farrowing rates, decreased litter size, stillbirths, and abortion^3,4^. Furthermore, during lactation, high temperatures exceeding 30 °C has been shown to impair sow milk synthesis^5^, as well as a reduction in some milk and colostrum components such as fatty acids and immunoglobulins due to limited blood supply to the udder and the reduction in feed intake^6^. Reduced immunoglobulins content in colostrum during the piglets’ first hours of life may pose a health challenge considering that colostrum milk offer piglets’ passive immunity, which is the primary source of protection against infections in newborns^7^. Additionally, intestinal hypoxia due to thermal load could reduce intestinal barrier function, leading to luminal lipopolysaccharide (LPS) from Gram-negative bacteria infusing into the blood circulation compromising the sow and fetus health as energy is apportioned to immune responses at the expense of core body reproductive functions^8^.

The significant production losses in pregnant and lactating sows associated with increased ambient temperatures calls for implementation of a novel feeding strategy to ameliorate these negative impacts in swine production. Traditionally, with respect to reduced ADFI and impaired nutrient uptake, provision of high energy concentrates feeds dense in nutrients has been adopted to mitigate the problem of low feed intake in sows under hot environmental conditions. However, this strategy during gestation is counterproductive as it may lead to overweight sows with accompanied parturition problems, depressed feed intake during lactation, and reduced milk production^9^.

The incorporation of high dietary fiber (DF) in sows’ diets during periods of high ambient temperatures has achieved beneficial results in recent years. DF are portions of plant derived feed that are resistant to enzymatic digestion in the small intestines of mammals, and fully or partially undergo microbial fermentation in the hindgut^10^. Based on water solubility, DF are categorized into two classes: soluble fiber (SF), such as gums, pectin, and inulin; and insoluble fiber (ISF), such as lignin and cellulose^7,11^. Many studies have demonstrated that increasing DF intake during pregnancy for one or more gestation-lactation cycles (reproductive cycles) when daily nutrient intake per sow is equalized among treatments can increase sow and litter performance^12,13^. However, other studies reported no impact or negative impacts on litter and sow performance when fiber was added to the gestation diet^14,15^. These discrepancies may be attributed to the type of dietary fiber supplemented during pregnancy. The ISF/SF ratio of a fiber resource may have an impact on diet consumption and play a key role in improving sow reproductive performance^7^. A previous study established that ISF/SF ratio had a significant impact on the health of sows and their offspring, with greater average piglet body weight (BW) and litter weight at weaning recorded when the ratio of insoluble to soluble fiber in the gestation diet was 3.89 compared to 5.59, 9.12, and 12.81 diets^16^. Therefore, for sow dietary fiber during gestation to be effective, there is a threshold on the ratio of insoluble to soluble ratio. It is important therefore, to comprehend the level of dietary fiber and the ratio of SF:ISF that could be sufficient to alleviate the impacts of heat stress in gestating sows. This study, aimed to determine the effects of DF level, the ratio of ISF/SF and their interaction if any on gut permeability, stress level, metabolites level in the gut and sow and litter performance under hot weather conditions.

## Results

### Respiratory rate and rectal temperatures

**Figure 1:**
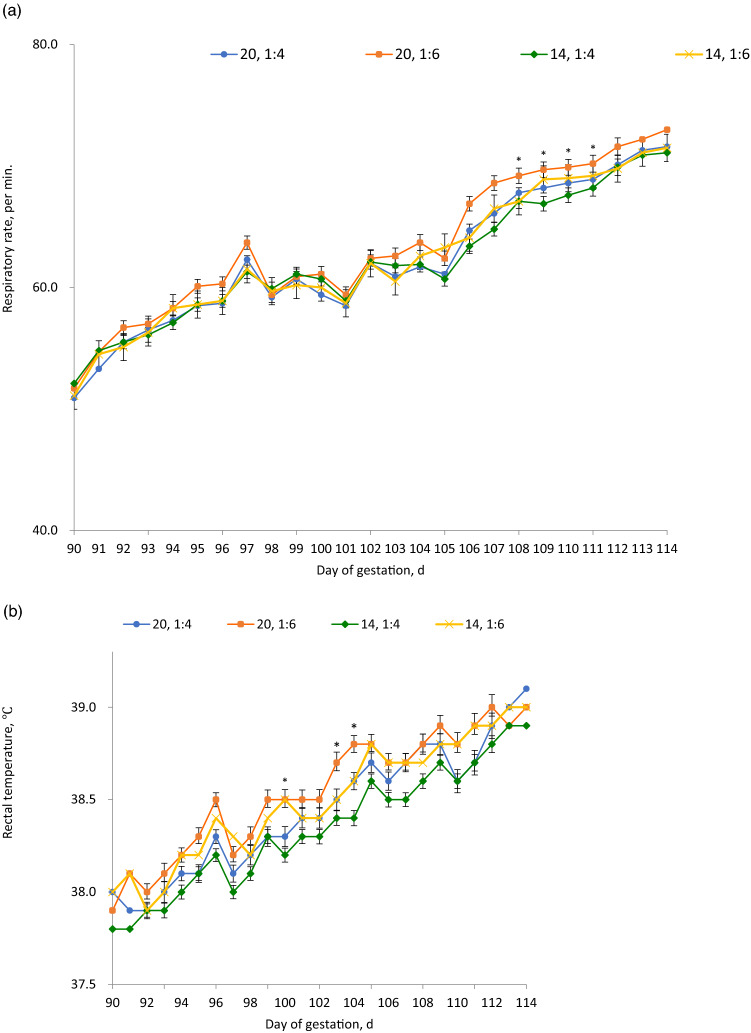
Effect of dietary fiber levels and soluble, insoluble dietary fiber ratio on: (**a**) respiration rate, (**b**) rectal temperature of sows during heat stress. Asterisks(*) indicate statistical significance (P < 0.05).

The respiratory rate was significantly higher (P < 0.05) from day 107–110 of gestation in the high SF groups compared to the high ISF groups. On the other hand, the rectal temperature was elevated (P < 0.05) in higher ISF-fed diets compared to the higher SF-fed groups on day 100, 103, and 104. No interaction was observed between fiber level and ratio of SF:ISF (Fig. 1a,b).

### Hair cortisol concentration

**Figure 2:**
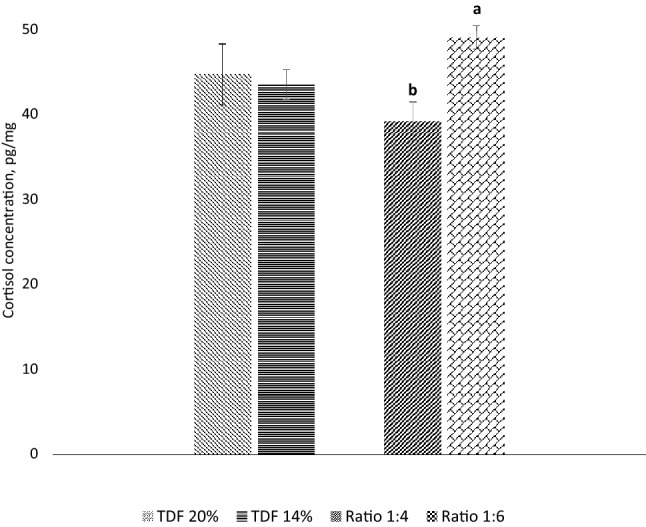
Effects of total dietary fiber (TDF) levels and soluble, insoluble dietary fiber ratio on hair cortisol concentration of sows. Superscript a, b means with different superscripts indicate statistical significance (P < 0.05).

Cortisol level was significantly lower (P < 0.05) in the high SF treatments compared to the high ISF treatments (Fig. 2).

### Serum superoxide dismutase and malondialdehyde levels

The concentration of superoxide dismutase (SOD) in the serum of sows tended to be higher (P = 0.056) in the higher SF- fed treatments compared to the higher ISF- fed treatments. There was tendency towards lower MDA concentration in serum (P = 0.069) in the high DF level treatments compared to the low level DF treatments (Table 1).

**Table 1:** Effects of total dietary fiber levels and soluble, insoluble dietary fiber ratio on serum oxidant, and antioxidant activity of sows.

Treatment	HH	HL	LH	LL	SEM	P-value		
Total DF, %	20	20	14	14		Fiber	Ratio	Fiber × ratio
SF:ISF	1:4	1:6	1:4	1:6				
SOD, U/ml	70.82	60.11	63.03	61.66	1.530	0.314	0.056	0.136
MDA, mmol/ml	1.68	1.69	1.79	1.81	0.120	0.069	0.822	0.864

*SEM* standard error of means, *SOD* superoxide dismutase, *MDA* malondialdehyde, *DF* dietary fiber, *SF* soluble fiber, *ISF* insoluble fiber.

^a,b^Means with different superscripts within rows are significantly different at (P < 0.05).

### Sows’ reproductive performance

The BF loss during lactation was higher (P < 0.05) in the high DF level fed groups than the low DF level groups. There was a tendency (P = 0.075) towards higher BF loss during lactation in the high ISF treatments compared to the high SF treatments. The ADFI was significantly higher (P < 0.05) in the high DF level diets than in the low DF level diets. The farrowing duration was significantly shorter higher (P < 0.05) in the high DF level treatments than in the low DF level treatments. Further, the farrowing duration was longer higher (P < 0.05) in the high ISF tretments compared to the high SF treatments.. The constipation index was significantly greater (P < 0.05) in the high SF-fed groups than the high ISF-fed groups. The WEI tended to be higher (P = 0.070) in the high DF level treatments compared to the low DF level treatments . There was no interaction observed between DF level and SF:ISF ratio (Table 2).

**Table 2:** Effects of total dietary fiber levels and soluble, insoluble dietary fiber ratio on sow reproductive performance.

Treatment	HH	HL	LH	LL	SEM	P-value		
Total DF, %	20	20	14	14		Fiber	Ratio	Fiber × ratio
SF:ISF	1:4	1:6	1:4	1:6				
**BW, kg**								
d 90	188.50	184.35	185.00	186.85	3.98	0.860	0.930	0.533
d 112	203.00	198.60	199.40	201.30	4.15	0.879	0.673	0.290
Gain during gestation	14.50	14.25	14.40	14.45	0.37	0.841	0.894	0.947
24 h postpartum	182.50	178.85	178.95	180.40	4.64	0.762	0.740	0.442
Weaning	167.35	163.15	163.55	164.60	4.29	0.701	0.608	0.394
Loss during lactation	15.15	15.70	15.40	15.80	0.40	0.827	0.554	0.925
**BF, mm**								
d 90	21.00	20.35	20.50	20.65	0.62	0.936	0.842	0.749
d 112	21.45	21.20	21.10	21.20	0.63	0.890	0.953	0.890
Gain during gestation	0.85	0.95	0.75	0.70	0.07	0.306	0.731	0.711
24 h postpartum	18.15	18.00	17.80	17.45	0.40	0.576	0.755	0.901
Weaning	15.90	15.40	14.95	14.15	0.66	0.192	0.437	0.857
Lossduring lactation	2.25^b^	2.60^ab^	2.85^ab^	3.30^a^	0.11	0.005	0.075	0.820
ADFI during lactation, kg/day	5.18^a^	5.14^a^	4.84^b^	4.81^b^	0.07	0.008	0.733	0.957
Farrowing duration, h	4.15^b^	4.55^ab^	4.56^ab^	4.89^a^	0.08	0.027	0.030	0.837
Constipation index^A^	2.66^a^	2.26^b^	2.47^ab^	2.17^b^	0.06	0.268	0.008	0.708
WEI	5.30	5.45	5.68	5.78	0.09	0.070	0.509	0.895

*SEM* standard error of means, *BW* body weight, *BF* backfat thickness, *ADFI* average daily feed intake, *DF* dietary fiber, *SF* soluble fiber, *ISF* insoluble fiber.

^A^A score value ranging from 0 to 5: 0 (absence of feces), 1 (dry and pellet-shaped), 2 (between dry and normal), 3 (normal and soft, but firm and well formed), 4 (between normal and wet; still formed, but not firm), 5 (very wet feces, unformed and liquid).

^a,b^Means with different superscripts within rows are significantly different at (P < 0.05).

Piglet weaning BW was significantly greater (P < 0.05) in the the high DF level treatments compared to the low DF level treatments and higher (P < 0.05) in the high SF-fed diets than in the high ISF-fed diets. The weaning litter weigh was higher in the high DF level groups than the lower DF level groups (P < 0.05). There was a tendency (P = 0.058) towards higher weaning litter weight in the high SF treatments compared to the high ISF treatments (Table 3).

**Table 3:** Effects of total dietary fiber levels and soluble, insoluble dietary fiber ratio on piglet performance.

Treatment	HH	HL	LH	LL	SEM	P-value		
Total DF, %	20	20	14	14		Fiber	Ratio	Fiber × ratio
SF:ISF	1:4	1:6	1:4	1:6				
**Litter size**								
Total born	13.30	13.30	12.90	12.80	0.28	0.430	0.930	0.792
Born alive	12.40	12.40	12.10	11.70	0.26	0.385	0.771	0.771
Weaned	11.40	11.40	10.90	10.50	0.37	0.226	0.320	0.911
Survivability of piglets, %	92.59	92.59	91.75	89.10	1.29	0.738	0.290	0.997
**Piglet weight, kg**								
At birth	1.31	1.27	1.29	1.28	0.02	0.700	0.212	0.488
At weaning	6.13^a^	5.76^ab^	5.60^b^	5.49^b^	0.12	0.002	0.048	0.264
**Litter weight, kg**								
At birth	17.53	16.74	16.48	16.41	0.37	0.226	0.790	0.554
At weaning	69.65^a^	63.21^ab^	61.24^ab^	57.04^b^	2.72	0.011	0.058	0.682

*SEM* standard error of means, *DF* dietary fiber, *SF* soluble fiber, *ISF* insoluble fiber.

^a,b^Means with different superscripts within rows are significantly different at (P < 0.05).

### Fecal short-chain fatty acids levels

The fecal concentration of; acetate, propionate, butyrate, isovalerate and the total SCFAs was significantly greater (P < 0.05) in the high SF treatments compared to the high ISF treatments. There was no interaction between the level of DF and the ratio of SF:ISF (Table 4).

**Table 4:** Effects of total dietary fiber levels and soluble, insoluble dietary fiber ratio on short-chain fatty acid in feces of sows.

Treatment	HH	HL	LH	LL	SEM	P- value		
Total DF, %	20	20	14	14		Fiber	Ratio	Fiber × ratio
SF:ISF	1:4	1:6	1:4	1:6				
**SCFA, µmol/g**								
Acetate	68.54^a^	65.16^ab^	66.58^ab^	62.35^b^	1.817	0.198	0.043	0.818
Propionate	15.41^a^	13.75^b^	15.14^a^	13.51^b^	0.329	0.442	0.001	0.953
Butyrate	8.34^a^	7.51^b^	8.07^a^	7.37^b^	0.339	0.551	0.029	0.852
Isobutyrate	3.35	3.28	3.27	3.29	0.124	0.794	0.825	0.700
Valerate	3.07	2.97	3.12	3.03	0.064	0.401	0.152	0.973
Isovalerate	5.14^a^	4.77^b^	5.15^a^	4.75^b^	0.125	0.966	0.004	0.903
Total SCFA	105.47^a^	99.93^b^	102.81^a^	95.92^b^	2.200	0.138	0.008	0.760

*SEM* standard error of means, *SCFA* short-chain fatty acid, *DF* dietary fiber, *SF* soluble fiber, *ISF* insoluble fiber.

^a,b^Means with different superscripts within rows are significantly different at (P < 0.05).

### Gut barrier biomarkers

Plasma zonulin concentration was significantly decreased (P < 0.05) in the high SF treatments than in the high ISF treatments. Fecal lipocalin-2 level was elevated in the high ISF-fed groups compared to the high SF-fed groups. There was a tendency toward lower serum LPS (P = 0.052) in the high SF treatments than in the high ISF treatments. The level of LBP in serum tended to be lower (P = 0.085) in the high SF-fed diets compared to the high ISF-fed diets (Table 5).

**Table 5:** Effects of total dietary fiber levels and soluble, insoluble dietary fiber ratio on plasma zonulin, fecal lipocalin, serum LPS and LBP levels of sows.

Treatment	HH	HL	LH	LL	SEM	P-value		
Total DF, %	20	20	14	14		Fiber	Ratio	Fiber × ratio
SF:ISF	1:4	1:6	1:4	1:6				
Zonulin, pg/ml	624.10^b^	740.03^a^	650.71^ab^	753.50^a^	31.83	0.533	0.002	0.838
Lipocalin-2, µg/g	26.93^b^	30.20^a^	26.50^b^	30.05^a^	0.357	0.423	> 0.001	0.683
LPS, ng/ml	73.96	78.67	74.44	80.47	2.678	0.674	0.052	0.807
LBP, ng/ml	28.56	30.17	29.06	31.37	1.104	0.447	0.085	0.754

*SEM* standard error of means, *LPS* lipopolysaccharides, *LBP* lipopolysaccharides-binding protein, *DF* dietary fiber, *SF* soluble fiber, *ISF* insoluble fiber.

^a,b^Means with different superscripts within rows are significantly different at (P < 0.05).

### Metabolites

**Figure 3:**
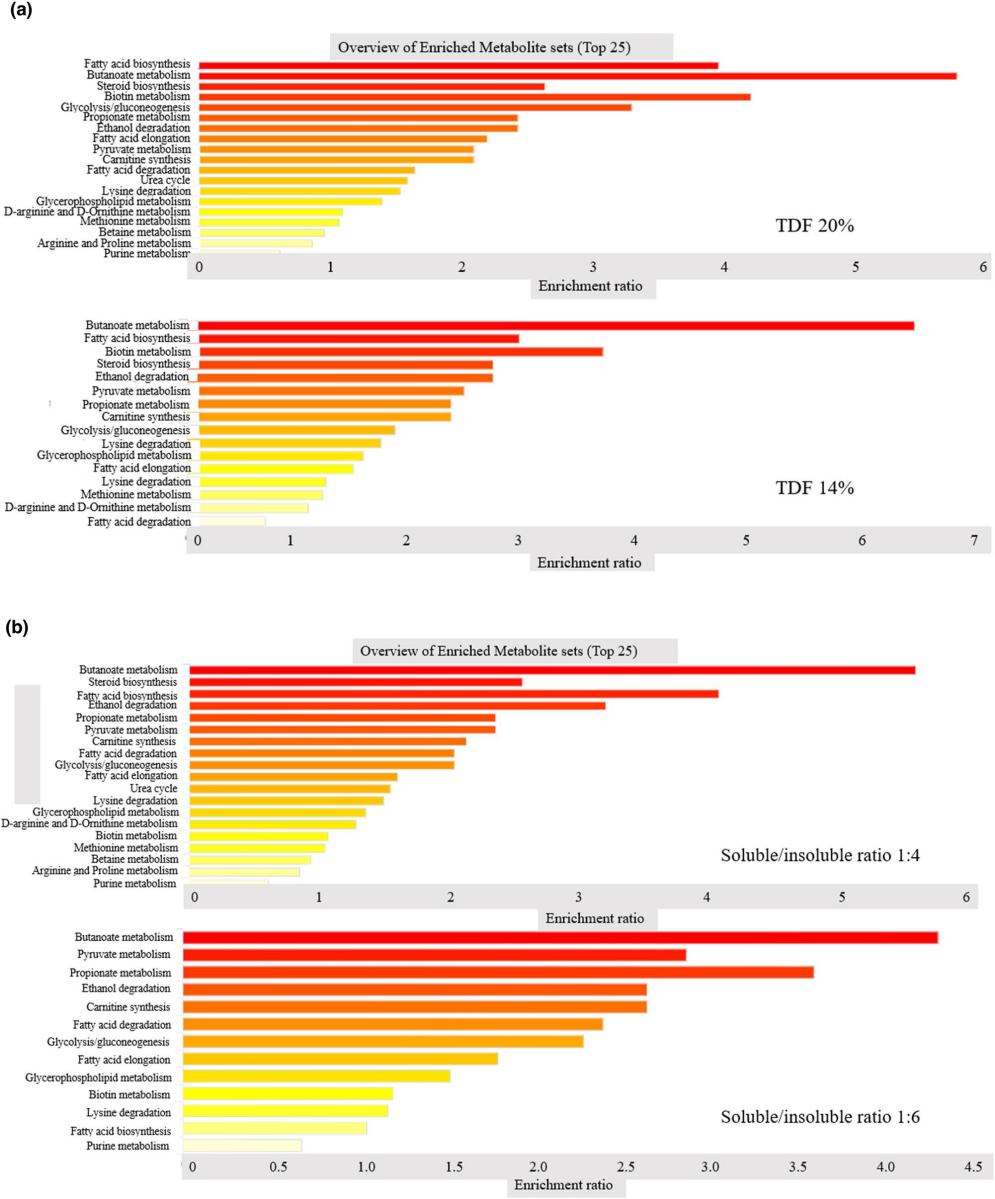
Effect of dietary fiber levels and soluble, insoluble dietary fiber ratio on significant metabolite sets accountable for class discrimination with variable importance projection (VIP) > 1 identified in the feces of sows fed different fiber levels during late gestation. (**a**) 20% vs. 14% ditary fiber level; (**b**) 1:4 vs. 1:6 soluble to insoluble fiber ratios.

**Figure 4:**
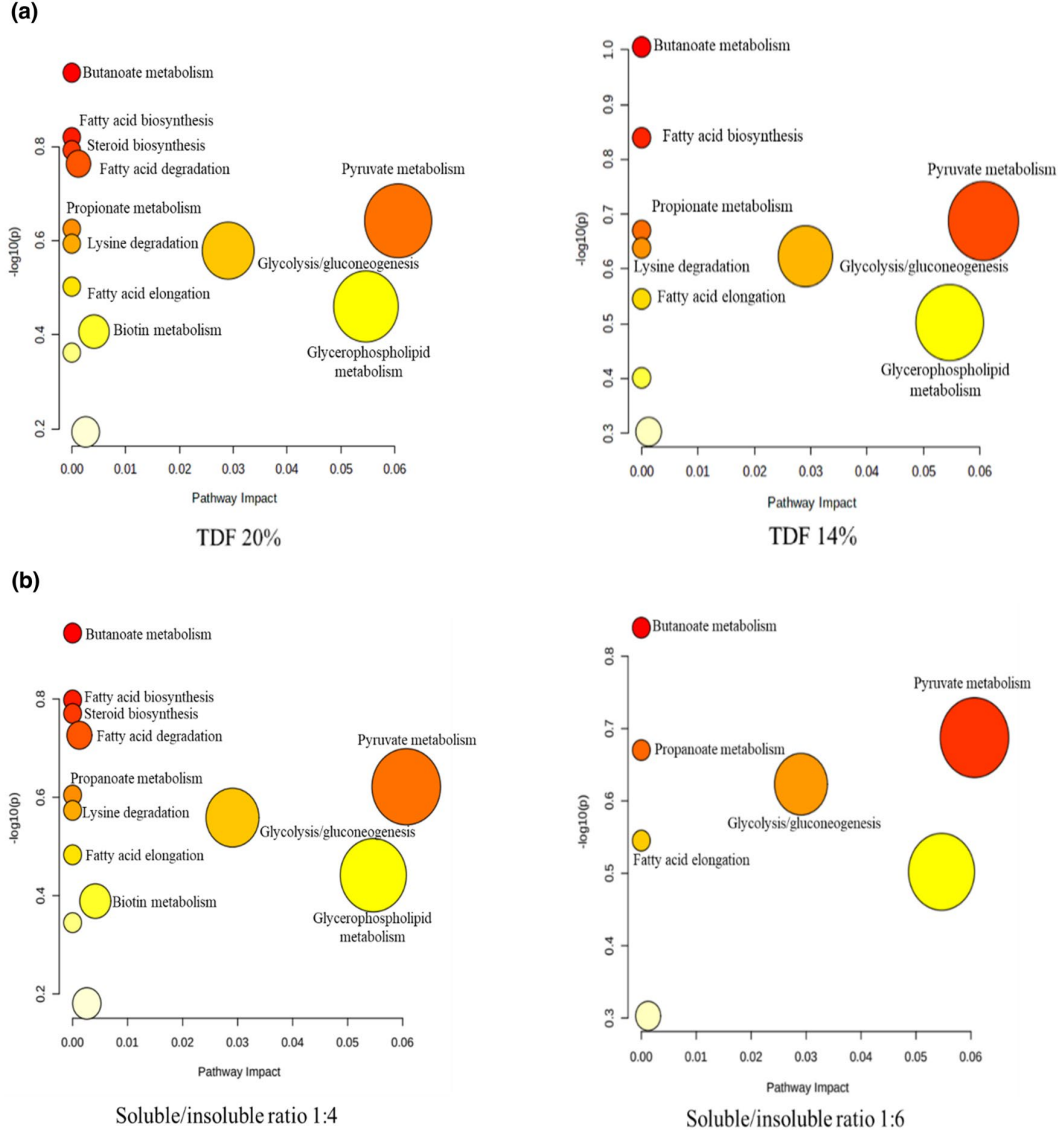
Effect of dietary fiber levels and soluble to insoluble dietary fiber ratio on metabolome view map of the differential metabolites (variable importance projection > 1, P < 0.05) identified in the feces of sows during late gestation. (**a**) 20% vs. 14% dietary fiber level; (**b**) 1:4 vs. 1:6 soluble to insoluble ratio. The x-axis represents the pathway impact, and the y-axis represents the pathway enrichment. The node color is based on its P-value, and the node radius is determined based on the pathway impact values. Larger sizes and darker colors represent higher pathway enrichment and impact values, respectively.

The variable importance projection VIP > 1 and p < 0.05 were utilized to determine the impacts of metabolite compounds on the variations. Metabolites including fatty acids lipids, carbohydrates, amino acids and organic acids in the sow’s feces were detected in multiple biochemical processes. The levels of Fatty acid biosynthesis, fatty acid elongation, urea cycle, arginine and proline metabolism, purine metabolism, betaine metabolism, Glycolysis/gluconeogenesis, and biotin metabolism were higher in sows fed on higher DF level (20%) than those on 14% DF level (Fig. 3a). On the other hand, butanoate metabolism, steroid biosynthesis, fatty acid biosynthesis, ethanol degradation, urea cycle, D-arginine and D-Ornithine metabolism, Methionine metabolism, Arginine and Proline metabolism, and Betaine metabolism were significantly increased in the higher SF:ISF diets (1:4) compared with the lower SF:ISF (1:6) treatments. The levels of pyruvate metabolism, and propionate metabolism were significantly increased in the lower SF:ISF (1:6) dietary treatments (Fig. 3b). On the basis of metabolite concentration changes, analysis of metabolic pathways identified steroid biosynthesis, biotin metabolism, and fatty acid degradation were significant in the 20% DF diets than the 14% treatments. Pyruvate metabolism was increased in the 14% DF level diets (Fig. 4a). Fatty acid biosynthesis, steroid biosynthesis, fatty acids degradation, lysine degradation, biotin metabolism was higher in the 1:4, SF:ISF treatments compared to the 1:6, SF:ISF diets. Glycolysis/ gluconeogenesis, and pyruvate metabolism were enhanced in the 1:6, SF:ISF treatments compared to the 1:4, SF:ISF diets (Fig. 4b).

## Discussion

Increased respiratory rates and vasodilation are some of the thermoregulatory mechanisms pigs use to increase the transfer of excess heat to the environment under high climatical conditions. High rectal temperature is occasioned by the diversion of blood from the viscera to the periphery in order to dissipate heat. On the other hand, increased respiratory rate increase lung evaporation and heat loss^17^. In this study, on day 114 of gestation, the sows' average respiratory rate averaged around 72mov/min, while the rectal temperature was at a high of 39 °C which is a sign of elevated body heat. Significant lower respiratory rates during days 107 to 110 of gestation and lower rectal temperature between d 100–104 of gestation were recorded in this study in the high DF level and SF-fed which is indicative of reduced body heat in these groups. Reduced respiratory rate lowers the thoracic muscle movements further lowering heat generation while reduced rectal temperature avoids cell apoptosis and organ damage.

Blood cortisol levels in mammals are frequently used as indicators of stress^18^. In our current study, cortisol level was significantly downregulated in the higher SF diets rather than the higher ISF diets which further illustrated lowered stress levels in the higher SF treatments. Additionally, oxidative stress levels can be determined by parameters such as MDA and SOD. In this study serum SOD tended to be higher in the higher SF treatments pointing towards improved antioxidant capacity and consequently declined stress^19^. Although we did not investigate blood cortisol levels, hair cortisol could be a better predictor of chronic stress as cortisol accumulates in the hair shafts during the experimental period. Besides, hair sampling being a non-invasive technique, especially during this stressful period in sows^20^. The mechanisms underlying the effects of dietary fiber on gestating sows' stress levels have not yet been fully understood, satiety may be linked to reduced cortisol.

Sow BW and BF during gestation did not differ among the groups in this study, which is consistent with earlier research showing that dietary fiber supplementation had no effect on BW and BF gain during gestation when energy was similar, regardless of fiber level or source^21^. Additionally, during gestation, the sow partitions most of its dietary energy towards the growth and development of the fetus rather than converting it to body reserves^22^. Sow BW at weaning was not affected by either the level of DF or the ratio of SF to ISF. We attributed this to the higher mobilization of lipids from fatty tissues to fulfill the demand for milk production^23^ since DF is less dense in nutrients. In our current study, we report reduced loss of BF in the higher DF diets and a lower tendency to BF loss in the higher SF diets. This could be due to enhanced fermentation giving rise to greater SCFAs especially acetic, butyric, and propionic acids, which are utilized in lactogenesis instead of body fat reserves^21^.

Lactational feed consumption is of paramount importance not only for sow subsequent productivity but also for the suckling litters’ growth performance. Low ADFI during lactation leads to increased mobilization of body reserves which has been linked to decreased sow’s reproductive performance^24^. Increased voluntary feed intake especially in primiparous sows could be significant in maintaining body condition as loss in body condition during lactation may result in reproductive failure^25^. In the present study, lactation feed intake was higher in the high dietary fiber diets compared with those fed on lower DF diets. Consistent with our results, Tan et al.^26^ reported increased ADFI during lactation when sows fed on the high fiber during gestation. We hypothesized that increased ADFI could be due to increased insulin sensitivity occasioned by the higher total fermentable fibers. During late pregnancy and lactation in sows, insulin resistance has been reported which negatively affects feed intake during lactation^27,28^. Additionally, high fiber diets during pregnancy potentially increase the size and capacity of the digestive tract, and the bulkiness of the diet makes it easier for sows to adjust to the significant rise in feed intake essential during lactation^29^.

Longer parturition time increases the danger of fetal hypoxia resulting in the number of stillborn piglets^30^. In this study, sows receiving high DF diet and higher SF-fed treatments had a shorter parturition duration. The shortened parturition time in the high fiber diets could be due to the reduction of stress responses in sows during parturition. A previous study observed improved postprandial satiety and reduced nervousness with higher DF intake during late gestation in sows and elimination of stereotypic behavior^31,32^ consequently reducing the farrowing duration. Furthermore, SF is readily fermented by the microbiota in the intestinal gut to yield SCFAs which provide extra energy for the sow to accelerate the parturition process^33^. Additionally, constipation may prolong farrowing duration through the hard feces acting as a physical barrier in the birth canal at parturition. As observed in this study, fecal score improved in the high DF-fed sows thereby decreasing constipation risk before and during farrowing.

Previous research has shown that sows fed an SF-rich diet during pregnancy had a higher rate of embryo survival and can farrow a greater number of total and live-born piglets than sows fed an ISF-rich diet^12^ which was attributed to the ability of the gut microbiota to easily ferment SF to yield metabolites such as secondary bile acids, serotonin, and SCFA that are beneficial to embryo growth and survival. Besides, increased intake of SF during gestation promotes beneficial gut bacteria while inhibiting the pathogenic ones hence improving sow health^16^ which could improve embryo survival. In this study, however, we did not observe increased litter size or increased litter weights at birth by feeding high fiber diet or ratio of SF to ISF. This could be due to the intervention time, as nutritional treatments were provided to sows in late gestation which may not be an effective time to adjust litter as litter size is partly determined during early pregnancy^34,35^. In this study, higher fiber addition in gestation diets resulted to higher average piglet BW at weaning. In agreement with a previous study, addition of fiber during pregnancy in sows improved piglets’ average BW at weaning^13,36^. We hypothesized that higher DF diets, could improve intesninal integrity and improve the villus height through nourishment of enterocytes by the increased SCFAs production from DF fermentationwhich in the hindgut. The improved intestinal morphology may increase the ability of intestine to absorb more nutrient during lactation, particularly the first weeks, leading to improved growth performance in suckling piglets in the higher DF treatments.

Dietary fibers by-bass host enzymatic digestion in the upper gut and undergo microbial fermentation in the colon and cecum to yield SCFAs, particularly acetic, butyrate and propionate^37,38^. In this study, higher quantities of butyric, acetic and propionic acids were observed in the high SF diets than in the higher ISF diets. Similar to our findings, higher production of SCFA in the hindgut in swine was reported with the incorporation of flaxseed meal compared to oat hulls as sources of SF and ISF respectively^39^. We hypothesized that the production of higher quantities of these three core SCFA (acetic, butyric, propionic) and total SCFAs in higher SF diets could be due to faster and extensive fermentation by microbiota in the gastrointestinal tract as compared to ISF^40^. This could be due to the higher solubility of the fermentable fiber fractions, which acts as substrates for microbial activity.

The integrity of the intestinal barrier is critical in the maintenance of intestinal homeostasis by excluding pathogens, endotoxins, and antigens from entering the mucosal lamina propria^41^. High zonulin level in plasma is a marker for impaired intestinal barrier. In this study, reduced zonulin and lipocalin-2 plasma levels in the high SF diets could have potentially improved intestinal barrier function hence reduced gut permeability as observed in the lower tendency of LPS and LBP in blood. We hypothesized that dietary SF could increase the abundance of anti-inflammatory bacteria while decreasing the abundance of gut proinflammatory bacteria by regulating the gut microbiota structure and SCFA production via fermentation of SFs. SCFAs could inhibit the nuclear factor kappa light chain enhancer of activated B cells (NF-κB) pathway and activate nuclear factor erythroid 2-related factor/ heme oxygenase-1 (Nrf2/HO-1) pathway which regulate intestinal cellular redox and protective antioxidant, thus reducing intestinal permeability resulting to decrease in blood endotoxins, lower oxidative stress, and decrease in inflammatory responses, potentially improving the health of sows and piglets.

In this study, metabolomic analysis revealed that in both higher DF and higher ratio of SF:ISF, biotin one of the vitamin B complex was upregulated in the fecal samples. Biotin is an important enzyme co-factor involved in vital metabolic pathways such as fatty acid synthesis. Further, butanoate metabolism which is prominently enriched in the higher SF:ISF treatments yields butyrate which is the main energy source that nourishes colonocytes hence rapid proliferation^18^ associated with improved gut barrier observed in the higher SF:ISF treatment groups in this study could be attributed to the enhanced butyrate. Steroids biosynthesis begins with acetic acid yielding cholesterol which is then converted to bile acids and steroid hormones such as estradiol and progesterone which have a huge impact in ovarian functions^42^. In this study, the high steroid biosynthesis observed in the high DF level and SF diets could be due to higher SCFAs generated from fiber fermentation, more especially acetic acid which the precursor for steroid synthesis. Additionally, the gluconeogenesis pathway was significantly enriched in the low DF level and high ISF-fed treatments which potentially could be an indicator of low SCFAs production leading to conversion of proteins and fats to glucose.

## Conclusions

The findings of the present investigation suggest that SF:ISF supplementation in sows during late gestation could lessen the harmful effects of heat stress. An increase in the SF:ISF ratio was associated with an increase in many metabolites in fecal samples. However, to assess the impact of dietary soluble and insoluble fibers on metabolite production and pathways at various phases of heat stress, more research is required. Further higher level of dietary fiber (20%) improved; ADFI during lactation, the constipation index, piglets' BW, and decreased the length of the farrowing process as well as BF loss during lactation. these positive characteristics help sows operate better in hot environments.

## Materials and methods

The experimental protocols were approved by the Kangwon National University, Chuncheon, Republic of Korea institutional animal care and use committee (KW-210503–6) as per the Regulations for the Administration of Affairs Concerning Experimental Animals of Republic of Korea.

### Animals and experimental design

**Figure 5:**
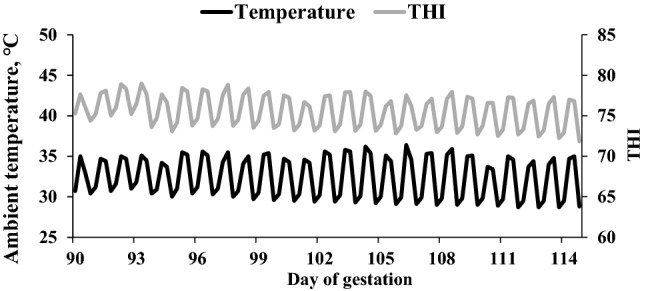
The ambient temperature and temperature humidity index (THI) during heat stress in sows during the experimental period. Ambient temperature (black line) and (THI) temperature-humidity index (grey line) during experimental period.

The experiment was undertaken in a commercial swine farm in Haman region in South Korea during the July–August summer of the year 2021, with average environmental temperature of 36.8 °C. Briefly, the sows were artificially inseminated two times after estrus onset, and pregnancy diagnosis and confirmation using ultrasound machine (AV 2100 V: Ambisea Tech. Corp, Shenzhen, China). At day 90 of gestation, A total of 40 Duroc × Landrace × Yorkshire multiparous sows [average parity = 3; approximately 197 ± 23 kg average body weight (BW)] were selected and allotted to one of the four treatments in a completely randomized experimental design to examine the effect of dietary fiber (DF) level, and ratio of insoluble fiber: insoluble fiber (SF:ISF). The treatment diets included 20% DF, 1:4 ratio of SF:ISF (HH), 20% DF, 1:6 ratio of SF:ISF (HL), 16% DF, 1:4 ratio of SF:ISF (LH), and 16% DF, 1:6 ratio of SF:ISF (LL). Each treatment included ten replicated pens composed of two sows from each parity (1, 2,3, 4, 5). One was housed per gestation stall (2.05 × 1.08 m) having concrete slatted floor. Ambient temperature in the gestation barn was averagely 32.2 °C (average day temperatures of 34.6 °C and night 29.8 °C). On day 100 of gestation, sows were relocated to the farrowing room and housed in individual farrowing creates with slatted floor until weaning at day 21 of lactation. A single-space feeder and a nipple drinker were installed in each farrowing crate, allowing for ad libitum access to feed and water. The corn-soybean meal-based basal diets were formulated in accordance with the National Research Council's 2012^43^ were provided from day 90 of gestation to weaning at day 21 of lactation to meet or surpass the nutritional needs of gestation and lactating sows. The formula, chemical composition, and analyzed dietary fiber characteristics of the experimental basal diets (as-fed-basis) are shown in Table 6. An infrared lamp was used to heat the piglet area within each farrowing crate. Temperature and humidity monitoring devices (Campbell Scientific Ltd., Shepshed, U.K) placed 1 m above the floor in the gestation and farrowing rooms respectively, were used to continuously measure temperatures and relative humidity and recorded every 5 min. Humidity and temperature were assessed using the temperature and humidity index (THI) as described by Dikmen et al.^44^. The THI was calculated using the following formula: THI = [(1.8 × T + 32) − (0.55 − 0.0055 × RH) × (1.8 × T − 26)] where RH is the  relative humidity in percent (%); T is the temperature in degree (^o^C). The temperature and THI during the gestation experimental period are shown in Fig. 5.

**Table 6:** Formula, chemical composition and analyzed dietary fiber characteristics of experimental basal diets (as-fed basis).

Total DF, %	20		14	
Soluble/insoluble ratio	1:4	1:6	1:4	1:6
Treatment	HH	HL	LH	LL
**Ingredient (%)**				
Corn	52.71	53.18	63.16	64.39
SBM dehulled	13.13	11.00	13.55	11.90
Wheat	3.51	14.98	2.16	8.44
Wheat bran	4.50	4.50	4.50	4.50
Sugar beet pulp	20.25	8.69	6.98	–
Soy hull	1.50	1.50	5.00	5.00
Soy oil	1.21	2.66	1.03	2.15
Salt	0.50	0.50	0.50	0.50
MDCP	1.12	1.02	1.16	1.05
Limestone	1.02	1.31	1.36	1.39
_DL_-Methionine (98%)	0.05	0.05	0.05	0.05
_L_-Lysine (78.8%)	0.05	0.14	0.08	0.14
_L_-Tryptophan (10%)	0.06	0.05	0.08	0.07
_L_-Threonine (98.5%)	0.08	0.10	0.07	0.09
Choline-chloride (50%)	0.06	0.07	0.07	0.08
Vitamin premix^a^	0.10	0.10	0.10	0.10
Mineral premix^b^	0.10	0.10	0.10	0.10
Phytase	0.05	0.05	0.05	0.05
Total	100.00	100.00	100.00	100.00
**Calculated composition**				
ME (Kcal/kg)	3,000	3,000	3,200	3,200
CP	13.40	13.40	14.30	14.30
Ca	0.69	0.69	0.70	0.70
Av.P	0.31	0.31	0.32	0.32
SID. Lys	0.58	0.47	0.56	0.50
SID. Met	0.20	0.24	0.23	0.24
SID. Met + Cys	0.48	0.49	0.47	0.56
SID. Thr	0.47	0.46	0.48	0.49
SID. Trp	0.14	0.13	0.13	0.14
ADF	5.05	5.26	3.95	4.09
NDF	16.23	16.61	11.59	11.98
TF	20.00	19.94	14.00	13.99
Soluble fiber	3.97	2.84	2.78	1.97
Insoluble fiber	16.03	17.10	11.22	12.02
SF/ISF ratio	1:4	1:6	1:4	1:6
**Analyzed dietary fiber characteristics**				
Total dietary fiber	20.06	19.99	13.96	13.97
Soluble fiber	3.96	2.85	2.82	1.99
Insoluble fiber	16.04	17.14	11.14	11.98
SF/ISF	4.05	6.01	3.95	6.02

*DF* dietary fiber, *SF* soluble fiber, *ISF* insoluble fiber, *ADF* acid detergent fiber, *NDF* neutral detergent fiber, *TF* total fiber.

^a^Supplied per kilogram of vitamin premix for 14% DF diets: 12,000,000 IU vitamin A, 2,400,000 IU vitamin D_3_, 132,000 IU vitamin E, 1500 mg vitamin K_3_, 3000 mg vitamin B_1_, 11,250 mg vitamin B_2_, 3000 mg vitamin B_6_, 45 mg vitamin B_12_, 36,000 mg pantothenic acid, 30,000 mg niacin, 600 mg biotin, 4000 mg folic acid. The vitamin contents were 6.7% lower in 20% DF diets.

^b^Supplied per kilogram of mineral premix for 14% DF diets: 80,000 mg Fe, 170 mg Co, 8500 mg Cu, 25,000 mg Mn, 95,000 mg Zn, 140 mg I, 150 mg Se. The mineral contents were 6.7% lower in 20% DF diets.

### Blood and fecal sample collection

Blood samples (8 mL) were taken by puncture of the ear veins of fasting sows and placed in vacuum tubes (5 mL) and heparinized tubes (5 mL) for analysis. The samples were centrifuged at 3000 for 15 min at 4 °C in vacuum tubes to extract serum samples, which were then stored at −80 °C for further analysis. Fecal samples were taken twice a day at 7:00 a.m. and 15:30 a.m, pooled, sealed in plastic bags, and frozen at −20 °C. Following sample collection, the fecal samples for each sow were thawed and pooled together, then dried for 72 h in a forced-draft oven (65 °C), ground through a 1-mm screen, and thoroughly mixed before a subsample was taken for chemical analysis.

### Rectal temperature and respiratory rate

A digital thermometer inserted 2 cm into the rectum was used to measure the rectal temperature every morning and evening from 0800 to 0900, and 1700 to 1800 respectively throughout the experimental period. The respiratory rate was equally recorded at the same time by counting the number of breaths/min through observation of the thoracic movement when sow is at rest on a lying position.

### Hair cortisol level

Cortisol levels in the hair were measured as previously described^45^. Hair samples were shaved from the foreheads of sows at day 90 and day 112 of gestation, respectively. Hair samples were gathered and kept in aluminum foil before being dried in polypropylene tubes (HM Hyundai Micro Co., Korea). To remove contaminations, samples were washed with 5 ml isopropyl alcohol three times and dried for seven days at room temperature (23 ± 1 °C). After drying, cortisol was extracted using a methanol dilution method and tested with ELISA kit according to the manufacturer's instructions (Cayman Chemical, Ann Arbor, MI).

### Superoxide dismutase and malondialdehyde blood plasma levels

Plasma SOD was determined by the method described by Sun et al.^46^. Total SOD (T-SOD) activity was determined using a previously reported indirect competition test between SOD and the indicator chemical, nitroblue tetrazolium, for superoxide generated by xanthine–xanthine oxidase^47^. T-SOD activity units were calculated by defining 1 unit as the amount of sample protein capable of inhibiting nitroblue tetrazolium reduction by 50% of maximum inhibition. The data were normalized to the amount of protein in the sample and represented as U/mg protein.Plasma lipid peroxidation was determined by the reaction of Malondialdehyde (MDA) with thiobarbituric acid (TBA) as described by Esterbauer and Cheeseman^48^. In brief, 1 mL of thawed plasma sample was added to 1 mL ethylenediaminetetraacetic acid [0.037 g ethylenediaminetetraacetic acid (EDTA) in 10 mL distilled water], 2 mL trichloroacetic acid (TCA) (3 g TCA in 30 mL distilled water), and 1 mL butylated hydroxytoluene (BHT) (0.2 g BHT in 10 mL ethanol) in a boiling tube. the mixture was then centrifuged at 1200 g for 15 min. 1 mL of the supernatant was then incubated in with 1 mL of TBA (0.134 g TBA in 20 mL distilled water) on a water bath at 90 °C for 20 min. A spectrophotometer (Bausch and Lomb Supertonic 70, Feldkirchen, Germany) was used to assess the absorbance at 532 nm wavelength after cooling.

### Sows’ reproductive performance

Sow weight and backfat thickness were measured at day 90 of gestation, within 24 h post-partum, and at weaning. Sow BW was measured by dynamic weighbridge method using electronic pig weigh scale (Qingdao AN-1T, Simei, Shanghai, China) as described by Kim et al.^36^. Backfat thickness was measured by ultrasonography (Sonolayer SAL-32B, Toshiba, Tokyo, Japan) at 65 mm from the midline at the last-rib level. Piglets were weighed individually at birth, and at weaning on day 21. Every morning, feed refusals were collected between 0700 and 0800, and fresh feed was immediately distributed. Feed consumption was obtained by getting the difference between feed allowance and the refusals collected the next morning. The weaning-to-estrus interval (WEI) for each sow was recorded as the number of days between weaning and onset of the next estrus.

### Fecal SCFAs concentration

The SCFA levels in fecal samples were determined using gas chromatography (GC) as described by Liu et al.^20^. The samples were separated using a TRACE 1310 GC with a flame ionization detector and analyzed on an HP-88 column (100-m length, 0.25-mm diameter, and 0.2-m film thickness from the producer). The following was the temperature schedule: 70 °C for 1 min, followed by an increase to 180 °C held at 25 °C for 1 min, 200 °C maintained at 10 °C for 1 min, 220 °C held at 2 °C for 10 min, and lastly 240 °C held at 20 °C for 6 min. The sample was run at a 20:1 split ratio and a 1.3 ml/min column flow rate. As a carrier gas, hydrogen is employed. The injector is set to 270 °C, while the detector is set to 290 °C.

### Gut barrier biomarkers

Sow serum and fecal samples were evaluated for lipopolysaccharide (LPS), zonulin, lipocalin-2, and binding protein LBP) using enzyme-linked immunosorbent assay technology (Nanjing Jiancheng Bioengineering Institute, Nanjing, China), inflammatory variables were assessed.

### Fecal metabolomics sample preparation and analysis

The amounts of metabolites in sow feces were measured using GC–MS. 100 mg fecal sample was transferred to 5-ml centrifuge tubes, mixed with 500 μL distilled water, and vortexed for 60 s, according to He et al.^18^. Then, as an internal quantitative standard, 1000 μL methanol was added and vortexed for 30 s. After 30 min of incubation on ice, the ultrasound machine was used to keep samples at 25 °C for 10 min. After that, the centrifuge procedure (5000 r/min; 5 °C; 15 min) was done. All the supernatants were dried and deposited in 2 mL centrifuge tubes. The dried samples were then added to 60 μL of methoxyamine solution in pyridine, vortexed for 30 s, before being reacted for 120 min at 37 °C. 60 μL trifluoroacetamide reagent (containing 1% FMCS) was added and centrifuged (5000 r/min; 5 °C; 15 min) for 90 min at 37 °C. The resultant supernatant was transferred to a sample vial then analyzed using an Agilent 7890A/5975C GC–MS (Agilent Technologies, Santa Clara, CA, USA).

### Statistical analyzes

The GLM procedure was used for the statistical analysis (SAS Inst. Inc. Cary, NC), based on a completely randomized design. Parity number was included as a fixed effect and initial BW as a covariate. Significant differences of (P < 0.05) among the treatment were considered statistically different using Turkey’s Honestly Significant Difference procedure. individual sow was considered as the experimental unit for all parameters. The metabolites were identified and normalized to (13C2)-myristic acid and stable isotope IS using the gathered raw data (http://srdata.nist.gov/gateway/, accessed on 6 February 2022). The software program SIMCAP + version 13.0 was used to carry out the statistical analysis (Umetrics, Umea, Sweden). The effect of heat stress on metabolic pathways and metabolite set enrichment analysis was determined as per (http://www.metaboanalyst.ca/faces/ModuleView.xhtml) online tool accessed on July 11, 2022^18^.

### ‘ARRIVE’ recommendation

The reporting in this manuscript conforms with the Animal Research: Reporting of In Vivo Experiments recommendations.

## Data Availability

The corresponding author can provide the datasets for this work upon reasonable request.

## References

[1] Seibert JT (2018). Characterizing the acute heat stress response in gilts: I. Thermoregulatory and production variables. J. Anim. Sci..

[2] Kim K (2019). Evaluation of high nutrient diets and additional dextrose on reproductive performance and litter performance of heat- stressed lactating sows. J. Anim. Sci..

[3] Nardone A, Ronchi B, Lacetera N, Bernabucci U (2006). Climatic effects on productive traits in livestock. Vet. Res. Com..

[4] Amavizca-Nazar A, Montalvo-Corral M, González-Rios H, Pinelli-Saavedra A (2019). Hot environment on reproductive performance, immunoglobulins, vitamin E, and vitamin A status in sows and their progeny under commercial husbandry. J. Anim. Sci. Tech..

[5] Renaudeau D, Quiniou N, Noblet J (2001). Effects of exposure to high ambient temperature and dietary protein level on performance of multiparous lactating sows. J. Anim. Sci..

[6] Cheng C (2018). Maternal soluble fiber diet during pregnancy changes the intestinal microbiota, improves growth performance, and reduces intestinal permeability in piglets. Appl. Environ. Microbiol..

[7] Li Y (2021). Effects of dietary fiber supplementation in gestation diets on sow performance, physiology and milk composition for successive three parities. Anim. Feed Sci. Technol..

[8] Lambert GP (2002). Selected contribution: Hyperthermia-induced intestinal permeability and the role of oxidative and nitrosative stress. J. Appl. Physiol..

[9] Long NM (2010). Maternal obesity and increased nutrient intake before and during gestation in the ewe results in altered growth, adiposity, and glucose tolerance in adult offspring. J. Anim. Sci..

[10] Shang Q, Liu H, Wu D, Mahfuz S, Piao X (2021). Source of fiber influences growth, immune responses, gut barrier function and microbiota in weaned piglets fed antibiotic-free diets. Anim. Nutr..

[11] Chawla R, Patil GR (2010). Soluble dietary fiber. Compr. Rev. Food Sci..

[12] Renteria-Flores JA, Johnston LJ, Shurson GC, Moser RL, Webel SK (2008). Effect of soluble and insoluble dietary fiber on embryo survival and sow performance. J. Anim. Sci..

[13] Wang YS (2016). Effects of inulin supplementation in low or high-fat diets on reproductive performance of sows and antioxidant defence capacity in sows and offspring. Reprod. Domest. Anim..

[14] Van der Peet-Schwering CMC (2003). Performance of sows fed high levels of nonstarch polysaccharides during gestation and lactation over three parities. J. Anim. Sci..

[15] Darroch CS, Dove CR, Maxwell CV, Johnson ZB, Southern LL (2008). A regional evaluation of the effect of fiber type in gestation diets on sow reproductive performance. J. Anim. Sci..

[16] Li Y (2019). Maternal dietary fiber composition during gestation induces changes in offspring antioxidative capacity, inflammatory response, and gut microbiota in a sow model. Int. J. Mol. Sci..

[17] D'Souza WN (2017). Differing roles for short chain fatty acids and GPR43 agonism in the regulation of intestinal barrier function and immune responses. PLoS ONE.

[18] He J, Zheng W, Lu M, Yang X, Xue Y, Yao W (2019). A controlled heat stress during late gestation affects thermoregulation, productive performance, and metabolite profiles of primiparous sow. J. Therm. Biol..

[19] Huang S (2020). Effects of dietary fiber sources during gestation on stress status, abnormal behaviors and reproductive performance of sows. Animals.

[20] Liu B (2018). Response of gut microbiota to dietary fiber and metabolic interaction with SCFAs in piglets. Front. Microbiol..

[21] Guillemet R (2007). Dietary fibre for gestating sows: Effects on parturition progress, behaviour, litter and sow performance. Animals.

[22] Hosseindoust A (2018). Effects of age at first breeding and dietary energy level during the rearing period of replacement gilts. J. Anim. Sci..

[23] Valros A (2003). Metabolic state of the sow, nursing behaviour and milk production. Livest. Prod. Sci..

[24] Xue JL, Koketsu Y, Dial GD, Pettigrew J, Sower A (1997). Glucose tolerance, luteinizing hormone release, and reproductive performance of first-litter sows fed two levels of energy during gestation. J. Anim. Sci..

[25] Tajudeen H (2022). Effects of various cooling methods and drinking water temperatures on reproductive performance and behavior in heat stressed sows. J. Anim. Sci. Technol..

[26] Tan C (2015). Effects of dietary supplementation of oregano essential oil to sows on oxidative stress status, lactation feed intake of sows, and piglet performance. Biomed. Res. Int..

[27] Mosnier E, Etienne M, Ramaekers P, Pere MC (2010). The metabolic status during the peri partum period affects the voluntary feed intake and the metabolism of the lactating multiparous sow. Livest. Sci..

[28] Tan C, Wei H, Ao J, Long G, Peng J (2016). Inclusion of konjac flour in the gestation diet changes the gut microbiota, alleviates oxidative stress, and improves insulin sensitivity in sows. Appl. Environ. Microbiol..

[29] Matte JJ, Robert S, Girard CL, Farmer C, Martineau GP (1994). Effect of bulky diets based on wheat bran or oat hulls on reproductive performance of sows during their first two parities. J. Anim Sci..

[30] Langendijk P, Plush K (2019). Parturition and its relationship with stillbirths and asphyxiated piglets. Animals.

[31] Sapkota A, Marchant-Forde JN, Richert BT, Lay DC (2016). Including dietary fiber and resistant starch to increase satiety and reduce aggression in gestating sows. J. Anim. Sci..

[32] Fabio ELB, Renata FNV, Silvio PM, Keila MRD (2014). Behavior and performance of sows fed different levels of fiber and reared in individual cages or collective pens. An. Acad. Bras. Cienc..

[33] Zhuo Y (2020). Inclusion of purified dietary fiber during gestation improved the reproductive performance of sows. J. Anim. Sci. Biotechnol..

[34] Krogh U (2015). Colostrum production in sows fed different sources of fiber and fat during late gestation. Can. J. Anim. Sci..

[35] Feyera T, Højgaard CK, Vinther J, Bruun TS, Theil PK (2017). Dietary supplement rich in fiber fed to late gestating sows during transition reduces rate of stillborn piglets. J. Anim. Sci..

[36] Kim KY (2020). Effects of free feeding time system and energy level to improve the reproductive performance of lactating sows during summer. J. Anim. Sci. Technol..

[37] Solà-Oriol D, Gasa J (2017). Feeding strategies in pig production: Sows and their piglets. Anim. Feed Sci. Tech..

[38] Cummings JH, Macfarlane GT (1991). The control and consequences of bacterial fermentation in the human colon. J. Appl. Bacteriol..

[39] Ndou SP, Kiarie E, Nyachoti CM (2019). Flaxseed meal and oat hulls supplementation: Impact on predicted production and absorption of volatile fatty acids and energy from hindgut fermentation in growing pigs. J. Anim. Sci..

[40] Jha R, Bindelle J, Rossnagel B, Kessel AV, Leterme P (2011). In vitro evaluation of the fermentation characteristics of the carbohydrate fractions of hulless barley and other cereals in the gastrointestinal tract of pigs. Anim. Feed Sci. Technol..

[41] Zhang S (2018). Effects of L-carnitine on reproductive performance, milk composition, placental development and IGF concentrations in blood plasma and placental chorions in sows. Arch. Anim. Nutr..

[42] Ashworth CJ, Toma LM, Hunter MG (2009). Nutritional effects on oocyte and embryo development in mammals: Implications for reproductive efficiency and environmental sustainability. Biol. Sci..

[43] National Research Council. *Nutrient Requirements of Swine*. 11th edn. (National Academies Press)

[44] Dikmen S, Hansen PJ (2009). Is the temperature-humidity index the best indicator of heat stress in lactating dairy cows in a subtropical environment?. J. Dairy Sci..

[45] Kim WS, Nejad JG, Roh SG, Lee HG (2020). Heat-shock proteins gene expression in peripheral blood mononuclear cells as an indicator of heat stress in beef calves. Animals.

[46] Sun YI, Oberley LW, Li Y (1988). A simple method for clinical assay of superoxide dismutase. Clin. Chem..

[47] Greenwald, R. A. *Handbook of Methods for Oxygen Radical Research*. 217–220. (CRC Press, 2018).

[48] Esterbauer H, Cheeseman KH (1990). Determination of aldehydes lipid peroxidation products: Malonaldehyde and 4-hydroxynonenal. Methods Enzymol..

